# An Atypically Located Hydatid Cyst

**DOI:** 10.1590/0037-8682-0589-2020

**Published:** 2021-03-08

**Authors:** Emine Parlak, Salim Başol Tekin

**Affiliations:** 1 Ataturk University, Faculty of Medicine, Department of Infectious Diseases and Clinical Microbiology, Erzurum, Turkey.; 2 Ataturk University, Faculty of Medicine, Department of Medical Oncology, 25050 Yakutiye, Erzurum, Turkey.

A 37-year-old man presented to a university hospital in Turkey due to seizures. Multiple lesions were detected in the brain through computed and in the liver and thorax through radiographic studies. Radiotherapy was applied to the brain for 10 days at another center on suspicion of malignancy. 

**FIGURE 1:**
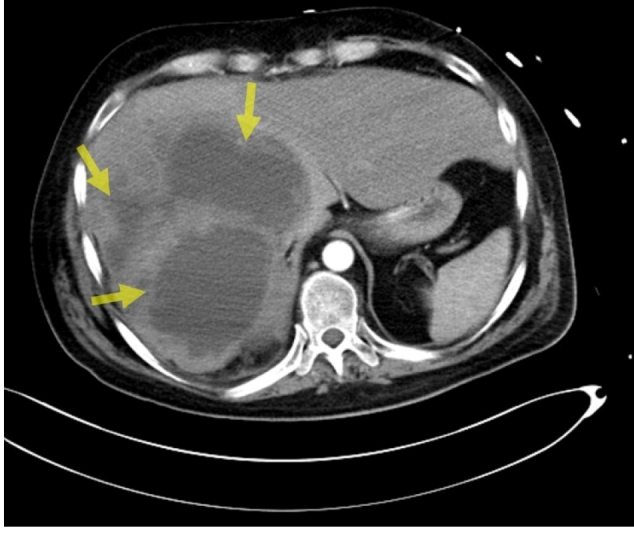
Multiple hepatic lesions.

**FIGURE 2:**
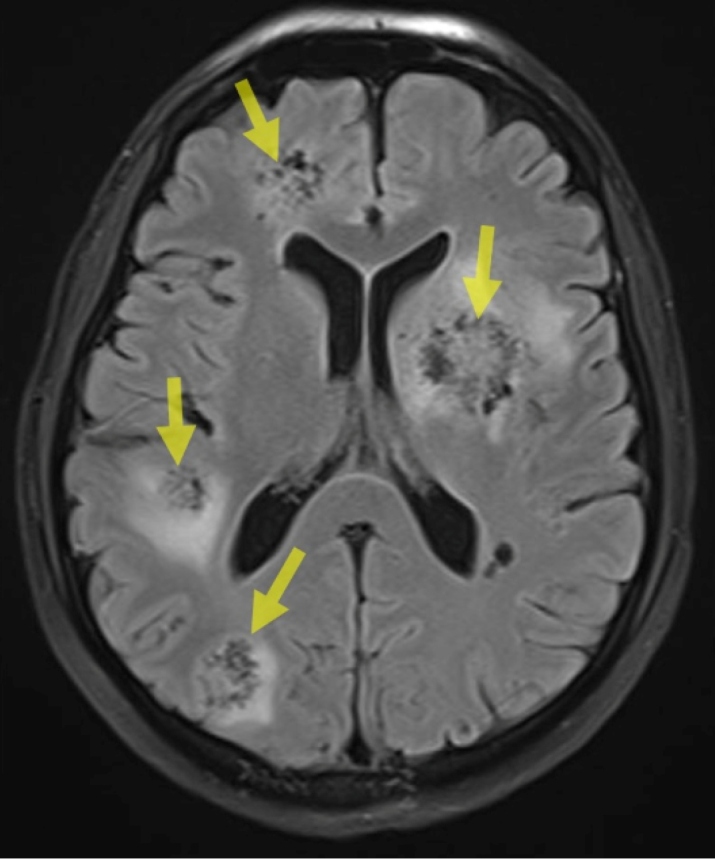
Multiple brain lesions.

**FIGURE 3:**
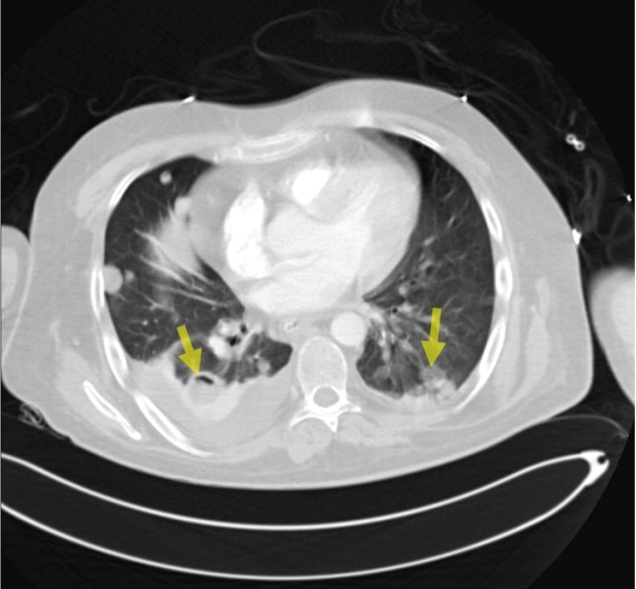
Multiple alveolar lesions.

The patient exhibited elevated body temperature and impaired consciousness; he was admitted to the internal disease intensive care unit for tests and treatment. He presented a poor general condition and lethargic consciousness. Principal preliminary diagnoses of metastatic tumors and hydatid cysts were considered. Multiple hepatic lesions, local invasion in the right adrenal gland, multiple pulmonary metastases, and local invasion in the upper pole of the right kidney were detected (Figure 1, Figure 2, and Figure 3). A liver biopsy was performed. The pathology report was compatible with alveolar echinococcosis. The ELISA test results were compatible with the diagnosis of echinococcosis (IgG = 2.4). Surgical treatment was not considered appropriate because of the widespread lesion. Albendazole treatment at 800 mg/day was started with a diagnosis of diffuse hydatid cyst. The patient lost consciousness and died. 

Hydatid cysts are an important zoonosis worldwide^1^. *Echinococcus granulosus* and *E. multilocularis* cause hydatid cyst disease and alveolar cyst disease, respectively^1^ ^,^ ^2^ Single organ involvement and a single cyst are more common. Treatment involves albendazole or mebendazole^3^. Hydatid cysts should be considered during differential diagnosis of patients with cystic masses in endemic regions. Community education is important. Despite being rare, systemic involvement may occur in addition to atypical localization. 
